# T1‐weighting in Steady‐State FLASH MRI–Diffusion Is Not Only Supportive but Mandatory for the Contrast

**DOI:** 10.1002/mrm.70443

**Published:** 2026-06-21

**Authors:** Simon Weinmüller, Deepak Charles Chellapandian, Jonathan Endres, Jochen Leupold, Florian Gritsch, Fabian Wagner, Rainer Schneider, Moritz Zaiss

**Affiliations:** ^1^ Institute of Neuroradiology, Uniklinikum Erlangen Erlangen Germany; ^2^ Department of Artificial Intelligence in Biomedical Engineering (AIBE) Friedrich‐Alexander‐Universität Erlangen‐Nürnberg Erlangen Germany; ^3^ Magnetic Resonance, Siemens Healthineers AG Erlangen Germany; ^4^ Medical Physics, Department of Diagnostic and Interventional Radiology University Medical Center Freiburg, Faculty of Medicine, University of Freiburg Freiburg Germany

**Keywords:** diffusion, gradient‐spoiling, RF‐spoiling, steady‐state, T_1_‐weighted FLASH, TR‐spoiling

## Abstract

**Purpose:**

FLASH imaging is widely assumed to produce a T_1_‐weighted steady‐state contrast using RF‐ and gradient‐spoiling. We observed substantial overestimation of CSF signals in simulations, when diffusion was neglected and realistic proton density was applied. This work investigates the role of diffusion in steady‐state FLASH contrast formation and its implications for simulation‐based modeling and measurement.

**Methods:**

FLASH sequences were simulated using phase graph simulations using a synthetic brain phantom with and without diffusion and realistic PD values to show the contrast change. The impact of neglecting diffusion in synthetic training data was evaluated using a segmentation network trained on simulated data and tested on in vivo measurement. Experimental validation of the contrast change was performed using a 3D‐printed brain phantom using silicone oil as a low‐diffusivity compartment.

**Results:**

Without diffusion, simulations showed CSF signal intensities higher than WM, resulting in a contrast change. Diffusion suppresses higher‐order echoes in long T_2_ tissues and is essential for achieving the T_1_‐weighted steady‐state contrast. A NN trained without diffusion fails to generalize to in vivo data and measurements with silicone oil compartments confirm the contrast changes in low‐diffusivity media.

**Conclusion:**

Diffusion is essential for realistic FLASH simulations of long T_2_ tissues such as CSF. Steady‐state FLASH contrast arises from the interplay of RF‐, gradient‐spoiling, and “multi‐TR‐relaxation‐spoiling” governed by T_2_‐decay and diffusion effects. For many quadratic phase cycling schemes, diffusion is required to obtain realistic T_1_‐weighted contrast in MR simulations and should not be neglected in simulations or simulation‐based deep learning applications.

## 1 Introduction

The FLASH sequence—or gradient‐ and RF‐spoiled gradient echo sequence—is one of the workhorses in clinical applications, since it enables T_1_‐weighted (T_1_w) contrast in the steady‐state regime [1, 2]. Gradient‐spoiling is not enough to achieve a T_1_w contrast, and an appropriate RF‐spoiling mechanism is necessary for short repetition time (TR) unbalanced gradient‐echo sequences [3]. The steady‐state signal S_SS_ of an ideally‐spoiled FLASH sequence is given by

(1) $$S_{\mathrm{ss}}=S_{0}\frac{1-\exp\left(-\mathrm{TR}/T_{1}\right)}{1-\exp\left(-\mathrm{TR}/T_{1}\right)\cos\left({\mathrm{rB}}_{1}\alpha \right)}$$

with repetition time TR, the flip angle α, the relative transmit magnetic field rB_1_, and the relaxation time T_1_. The scaling factor S_0_ is proportional to $\sin\left({\mathrm{rB}}_{1}\alpha \right)$, the proton density (PD) and has contributions from $T_{2}^{*}$T_2_*.

Ideal‐spoiling means in this context the elimination of higher‐order echoes and sole contribution of the free induction decay (FID) path to the signal. However, the complete elimination of T_2_‐weighted paths like spin echoes or stimulated echoes is hard to achieve even if RF‐spoiling is used. Zur et al. [4] and Sobol et al. [5] introduced the concept of quadratic phase cycling for the steady‐state following:

(2) $$\phi \left(n\right)=0.5\cdot \Psi \left(n^{2}+n+2\right)$$

where Ψ is the phase difference increment and ϕ(n) the phase of the *n* ^th^ pulse.

**FIGURE 1:**
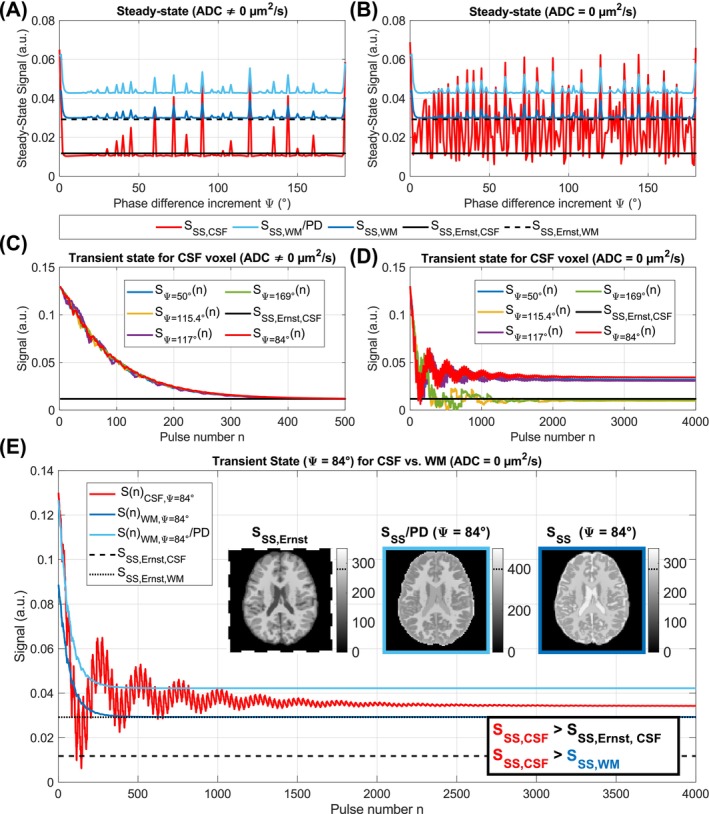
Steady state signal for CSF and WM voxel for different quadratic phase cyclings with phase difference increment Ψ. Diffusion plays an important role for steady state contrast as can be seen in the difference of the simulation result with diffusion (A) and without diffusion (B). Transient state is shown with (C) and without (D) diffusion for Ψ = 50°, 84°, 115.4°, 117° and 169° phase difference increment. The steady‐state CSF‐WM contrast is inverted (S_SS,CSF_ > S_SS,WM_) when ignoring diffusion effects for a phase difference increment of Ψ = 84° (E). The sequence parameters are α = 8°, TE = 2.0 ms, and TR = 4.0 ms; phantom parameters are for WM (T_1_ = 0.84 s, T_2_ = 0.075 s, D = 0.65 × 10^−3^ mm^2^/s, PD = 0.7) and CSF (T_1_ = 4.14 s, T_2_ = 1.64 s, D = 3.18 × 10^−3^ mm^2^/s, PD = 1.0).

The efficiency of this quadratic phase cycling is still strongly dependent on the specific phase difference increment Ψ, as displayed in (Figure 1A,B). The choice for the actual phase difference increment is done by matching the plot of Ψ with the ideally‐spoiled T_1_w contrast (Equation 1). Many different phase difference increments have been published to approximate ideal‐spoiling, such as 40.5° [6], 50° [7], 72° [8], 96.5° [9], 115.4° [10], 117° [4], [8], 118.2° [7], 121.8° [7], 129.3° [11], 144° [12], 150° [13], and 169° [6], [14] for the steady‐state regime or 84° [15] and optimized RF phase trains leaving the quadratic phase cycling regime [16] for the transient state. The overall goal of all proposed phase difference increments is the elimination of T_2_ contribution to the signal. However, the elimination of T_2_ contribution to the steady‐state signal is only approximately fulfilled [17].

In addition, the influence of diffusion on the RF‐spoiled signal is considered in the literature: Yarnykh [14], Heule et al. [18] and Corbin/Callaghan [12] examined the influence of imperfect spoiling on T_1_‐determination. Nehrke [11] investigated the influence of a diffusion damping factor on actual flip angle imaging. Leupold et al. [6] investigated how accurate different quadratic phase cyclings guarantee the T_1_w contrast, in particular for different diffusion strengths. They demonstrated that a phase difference increment of 169° shows T_1_w contrast for very low diffusion coefficients, concluding that diffusion is a helpful contributor for actual spoiling.

The simple and insightful results we want to present are image contrast simulations in the brain with and without diffusion. We observed that diffusion not just supports spoiling, but is mandatory to achieve the known T_1_w FLASH steady‐state contrast. Many known phase difference increments of quadratic phase cycling approaches lead—together with realistic PD values and no diffusion—to an overestimation of the CSF signal and to a contrast change in simulation. The assumed T_1_w contrast emerges from a complex interplay of gradient‐ and RF‐spoiling, and a “multi‐TR‐relaxation‐spoiling” of T_2_ and diffusion processes: Sufficient T_2_ relaxation and diffusion‐induced dephasing effects suppress higher‐order echo pathways so that the resulting FLASH contrast becomes T_1_‐weighted. If the latter spoiling mechanism is not present, the FLASH steady‐state contrast significantly depends on T_2_.

As either T_2_ decay or diffusion damping effectively suppresses these unwanted signal pathways in any healthy in vivo tissues, this effect gets only relevant for imaging scenarios of oils or other tissues with lower D values (e.g., in pore/capillary materials with very low diffusion lengths [19, 20, 21]), such as in studies involving phantoms or contrast media [6, 22, 23]. To illustrate the predicted contrast change for different phase difference increments experimentally, we performed measurements using a 3D‐printed brain phantom filled with silicone oil.

The contrast change becomes highly relevant in the context of simulation‐based modeling. Simulations are more and more used as physics‐informed models for reconstruction [24, 25], to generate synthetic data for network training [26], e.g., segmentation tasks [27], or dictionary‐based quantification [28, 29, 30, 31, 32, 33]. A model using data from simulations not considering diffusion effects could lead to wrong predictions. In this work, we demonstrated that a segmentation network trained on simulation data generated without diffusion can fail when applied to in vivo measurement.

Within this work we (i) investigate the influence of diffusion on steady‐state FLASH contrast formation, (ii) analyze higher‐order echo pathway contributions, (iii) show the impact of neglecting diffusion in simulation‐based neural network training, and (iv) demonstrate the contrast inversion experimentally in in vitro measurements.

## 2 Methods

We simulated a FLASH sequence on a brain phantom to evaluate its steady‐state contrast characteristics. FLASH sequences were defined in PyPulseq [34, 35] with the following parameters: α = 8°, TE = 2.0 ms, and TR = 4.0 ms, bandwidth of 800 Hz/px, FOV = 200 × 200 mm^2^, matrix size 96 × 96 with gradient spoiler in read direction with a moment corresponding to 3 times the maximum k‐space gradient moment (36 mT*ms/m), and quadratic phase cycling with phase difference increments that vary over the experiments. We compare the widely used 117° phase difference increment with 169°. The steady‐state regime is guaranteed by applying 4000 dummy pulses prior to signal acquisition. The seq‐files passed the Pulseq sequence check and can be executed on a clinical MR scanner. They are available upon request.

For MR simulation we used a phase distribution graph (PDG) [36] simulation, which describes the signal behavior of MR sequences, allowing both encoded and unencoded signal analysis. The simulation requires a 3D object defining the MR parameters (PD, T_1_, T_2_, T'_2_, rB_1_, ΔB_0_, and the isotropic diffusion constant D) to simulate the MR signal. A brain phantom is built from the data provided by the BrainWeb database [37]. Segmented maps for GM, WM, and CSF are filled with values for the various physical properties [38, 39, 40]. The summation of above‐mentioned values, weighted by their contribution to every voxel as given by the BrainWeb data, results in the input virtual phantom used by the simulation. Constant field inhomogeneities ΔB_0_ = 0 Hz and rB_1_ = 1 were assumed for all experiments. A comparison to an isochromat simulation and accuracy analysis of PDG is shown in the Supporting Information.

For single voxel simulation a voxel phantom is defined with one non‐zero voxel in the isocenter and with one set of MR parameters like WM (T_1_ = 0.84 s, T_2_ = 0.075 s, D = 0.65 × 10^−3^ mm^2^/s, PD = 0.7) or CSF (T_1_ = 4.14 s, T_2_ = 1.64 s, D = 3.18 × 10^−3^ mm^2^/s, PD = 1.0). The phantoms can be modified by assigning arbitrary values to any MR tissue parameter, for example changing the diffusion coefficient explicitly to zero for a simulation without diffusion effects. All PDG simulation results agreed with EPG simulations by Stöcker et al. [41, 42] and Leupold et al. [6].

For closer analysis of the MR signal composition, we used the τ‐dephasing unnormalized latent signal plot provided by the phase distribution graphs [36]. The latent signal provides insight, which echoes can contribute to the signal in the current or any later repetition. For a gradient‐spoiled FLASH sequence, this would be rephased echoes once restored back into the z‐magnetization. The ideally‐spoiled FLASH signal, consisting only of the FID path, would correspond to one line at τ=0. Thus, every intensity at the latent graph reflects unwanted signals that can interfere with each other, especially with the FID signal. These originate from higher echoes given by τ≠0., which indicate the dephasing time of transverse magnetization.

To investigate the importance of diffusion in the simulation, we trained a segmentation network (2D nnU‐Net) [43] using two different training datasets: (i) training data generated with diffusion explicitly included in the PDG simulation, and (ii) training data generated without considering diffusion. Each network type was trained for phase difference increments of 117° and 169°. In total, 228 synthetic 2D images were simulated using the PDG simulation, corresponding to 12 distinct slices from 19 BrainWeb [37] phantoms. These images were further augmented with Gaussian noise and bias fields, resulting in a total of 684 training samples. Exemplary simulated training data are shown in Figure S5. The underlying sequence was a steady‐state FLASH sequence with 4000 dummy pulses, flip angle α = 8°, TE = 2.56 ms, and TR = 5.7 ms, bandwidth of 400 Hz/px, FOV = 220 × 220 mm^2^, matrix size of 220 × 220 with gradient spoiler in read direction with a moment corresponding to 3 times the maximum k‐space gradient moment (36 mT*ms/m). The slice thickness was 2 mm, resulting in a spatial resolution of 1 × 1 × 2 mm^3^. The target labels were derived from the segmentations provided by BrainWeb [37]. Network performance was evaluated using in vivo measurement data. To avoid flow artifacts, a 3D volume with identical sequence parameters, but 36 slices was acquired in vivo on a 3 T scanner Magnetom Cima. X (Siemens Healthineers AG, Erlangen) after obtaining ethical approval. In contrast to a single 2D acquisition, this 3D approach ensures that the adjacent slices are also in the steady state, preventing fresh magnetization from neighboring slices from influencing the signal in the current slice.

A 3D‐printed brain phantom (see Figure 4A,B) was used to experimentally validate the simulated contrast behavior. Following [5], the ventricles were filled with silicone oil (PD ≈ 1.0, T_1_ ≈ 630 ms, T_2_ ≈ 125 ms, D ≈ 0.03 × 10^−3^ mm^2^/s), representing a low‐diffusivity medium. The GM (PD ≈ 0.35, T_1_ ≈ 140 ms, T_2_ ≈ 90 ms, D ≈ 2.3 × 10^−3^ mm^2^/s) and WM (PD ≈ 0.4, T_1_ ≈ 100 ms, T_2_ ≈ 50 ms, D ≈ 2.0 × 10^−3^ mm^2^/s) compartments were filled with water doped with CuSO_4_. The concentrations were adjusted to preserve the physiological ordering of T_1_ relaxation times between GM, WM, and CSF. PD differences of water and oil were implicitly controlled by varying the fill level of the respective compartments. A steady‐state FLASH sequence was acquired at a Cima. X scanner (Siemens Healthcare, Erlangen) with two different phase difference increments (Ψ = 117° and Ψ = 169°). Imaging parameters were: α = 5°, TE = 1.23 ms, TR = 2.72 ms, FOV = 220 × 220 × 20 mm^3^, matrix size of 100 × 100, bandwidth = 2000 Hz/pixel. A gradient spoiler was applied in the readout direction with a moment corresponding to the maximum k‐space gradient moment (12 mT*ms/m). A partial Fourier factor of 6/10 was used in read direction to reduce TR.

## 3 Results

The steady‐state signal was simulated for both a WM and CSF voxel in (Figure 1A,B) for different phase difference increments in steps of 1°, both with and without diffusion effects. Local sharp peaks are a known effect of various coherence pathways [8]. Figure 1A,B is already known from the literature [4, 14], however, we consider the PD factor of PD_CSF_/PD_WM_ = 1/0.7 = 1.43 here. In general, in a WM voxel, diffusion has only a minor influence on the steady‐state signal, since higher‐order echoes have already decayed strongly due to the short T_2_.

In contrast, diffusion plays a major role in CSF voxels. With diffusion included (Figure 1A), the same local sharp peaks as for WM are visible. Diffusion effectively suppresses higher‐order echoes even for longer T_2_ values. Without diffusion, however, a more random fluctuation and an overestimation of the observed steady state signal occur along the Ψ‐dimension. The steady‐state signal of a CSF voxel can exceed the signal of a WM voxel, especially if the effect of PD is properly addressed (peaks in Figure 1B).

Certain phase difference increments can produce a similar contrast that remains robust even for small diffusion effects, for example a phase difference increment of 169°. This choice has already been indicated by Leupold et al. [6]. With diffusion, different Ψ show Epstein fluctuations [15, 16] during the approach from transient state to the same steady‐state, as visualized in (Figure 1C). The fluctuations are stronger and certainly reflect no ideal exponential decay when no diffusion is considered for simulation (Figure 1D). The transient decay takes much longer without diffusion effects and its substructures depend on Ψ. For a phase difference increment of 84° the steady‐state signal is inverted (S_CSF_ > S_WM_) when ignoring diffusion effects, while a phase difference increment of 169° keeps the Ernst T_1_w contrast (S_CSF_ < S_WM_) (Figure 1E). In both the line plots and the images in (Figure 1E) the weakening of T_1_‐weighting is already visible when PD is not considered. The contrast inversion and complete loss of the T_1_‐weighting feature, however, is only revealed when correct PD values are used in the no diffusion case.

Thus, we conclude that diffusion is not only supportive but mandatory for the T_1_‐weighted contrast of FLASH.

**FIGURE 2:**
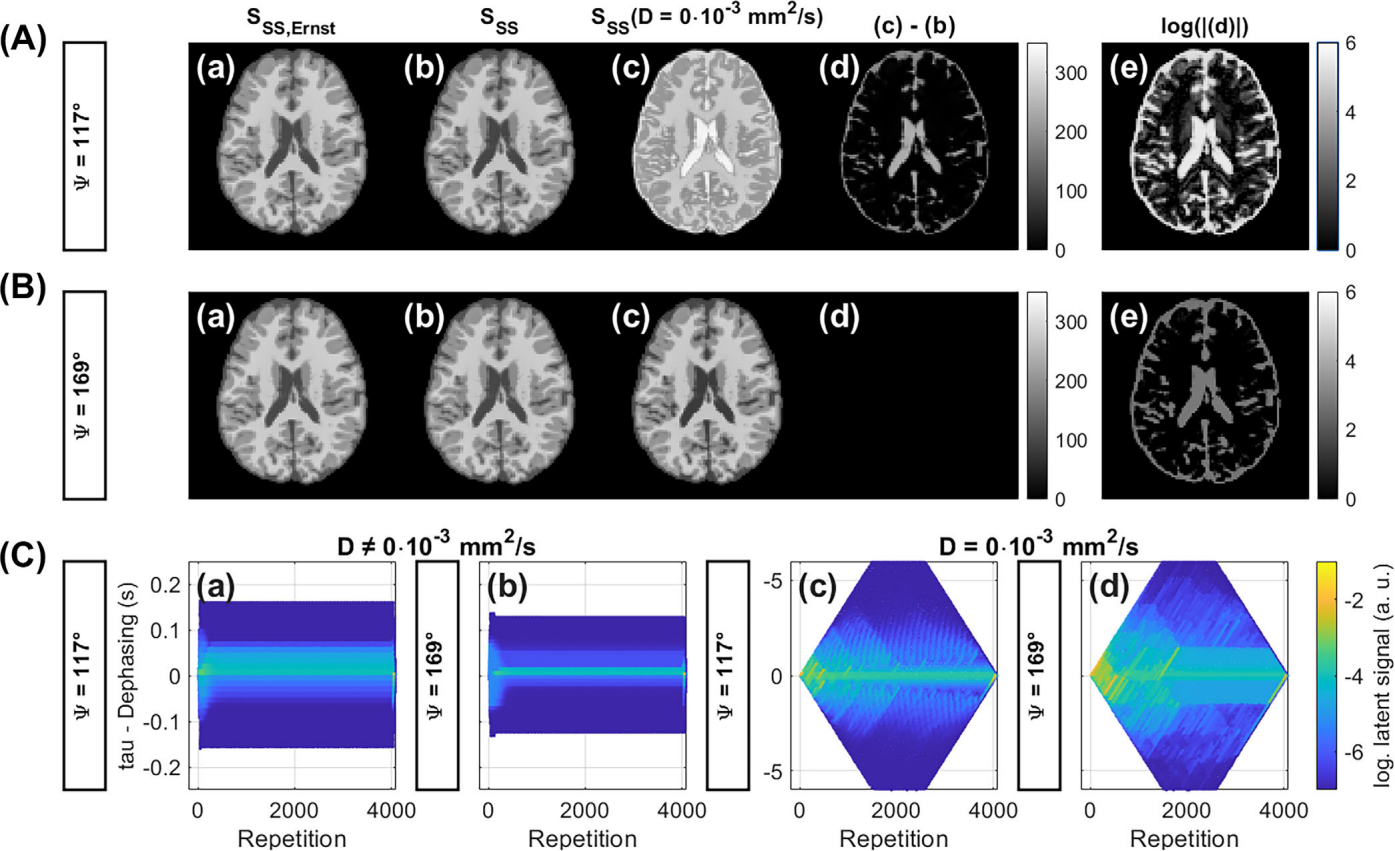
Steady‐state contrast for ideally spoiled FLASH sequence (a) and for a quadratic phase cycling with phase difference increment of 117° (A) and 169° (B) for different phantom configurations (b, c). In (b) the standard BrainWeb phantom including diffusion is used, while in (c) the diffusion map is set to 0 mm^2^/s, (d) shows the difference between (c) and (b). Same windowing is used in (a–d). Sequence parameters are α = 8°, TE = 2.0 ms, and TR = 4.0 ms. The latent signal for the simulation for a CSF voxel is shown in (C) for a simulation with and without diffusion. Signals below 10^−7^ are considered negligible for the final signal and are not shown.

Comparing the image contrasts in more detail (Figure 2), both the Ernst angle simulation and the simulation with realistic diffusion and phantom tissue properties correspond to the T_1_‐weighted FLASH contrast of an actual acquired image (Figure 2Aa, b). The same is observed for Ψ = 169° (Figure 2Ba, b). The previously discussed contrast change for Ψ = 117° (Figure 2Ac) is not observed for Ψ = 169° (Figure 2Bc). A signal increase is mainly visible in CSF with long T_2_ values (Figure 2A, Bd). However, GM already shows a slight increase (Figure 2A, Be). Further analysis of different scaling factors of diffusion and T_2_ for different phase difference increments is shown in Figure S2. To further understand this invariance of the setting Ψ = 169°, the unnormalized latent signal metric of the phase distribution graphs [36] for a CSF voxel is plotted in Figure 2C. For standard diffusion, both latent signal graphs look very similar (Figure 2Ca, b) as higher phase paths are depleted by diffusion and T_2_. Without diffusion, a higher number of stronger τ‐dephased states contribute to the measured signal for both investigated Ψ values (Figure 2Cc, d). The phase difference increment of 169° leads to a wider and more homogenous distribution of states. To explain the T_1_w contrast without diffusion, it is most plausible that for Ψ = 169° higher‐order states cancel each other out more efficiently.

**FIGURE 3:**
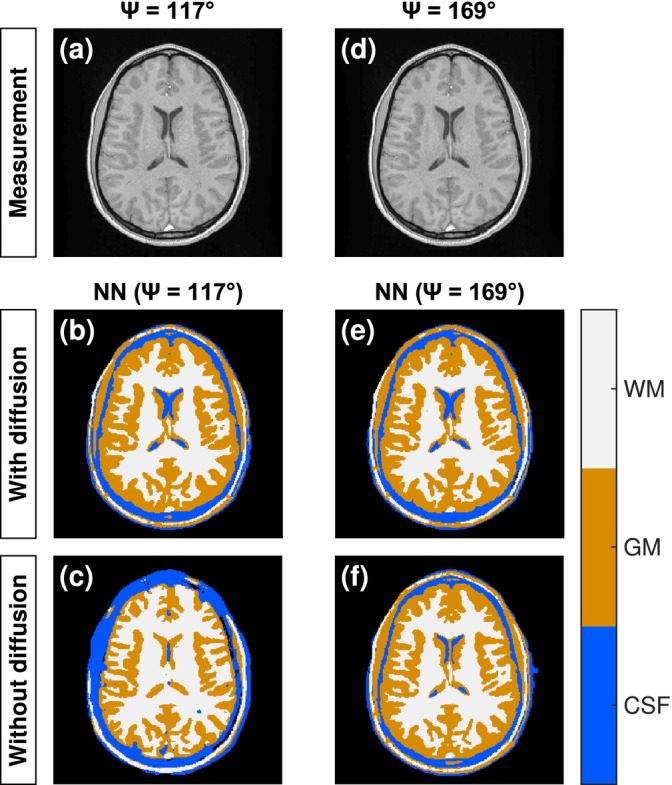
Comparison of measured input data and segmentation results for Ψ = 117° and 169°. (a, d) Measured steady‐state FLASH magnitude image used as network input. (b, c and e, f) Corresponding tissue segmentations for WM, GM, and CSF. (b, e) Segmentations obtained from networks trained with diffusion for both phase difference increments. (c, f) Segmentations obtained from networks trained without diffusion for both phase difference increments. Exemplary training data is shown in Figure S5.

A segmentation network was trained on simulated data generated with and without diffusion to directly assess its impact on downstream performance. During training, the exponential moving average pseudo Dice of all networks exceeded 0.95. Networks trained with diffusion show a good segmentation result for both phase difference increments of 117° and 169° (Figure 3b,e), compared to networks trained on data generated without diffusion (Figure 3c). In particular, the network fails for ψ = 117° (Figure 3c) in the CSF compartment, as expected. In the corresponding simulated training data, CSF exhibits increased signal intensity, which is not observed in the measurement data. This mismatch between simulated contrast and in vivo signal behavior leads to systematic misclassification of CSF. The phase difference increment of 169° is more robust to diffusion‐related contrast variations of the steady‐state contrast, which translates to the segmentation performance (Figure 3f).

**FIGURE 4:**
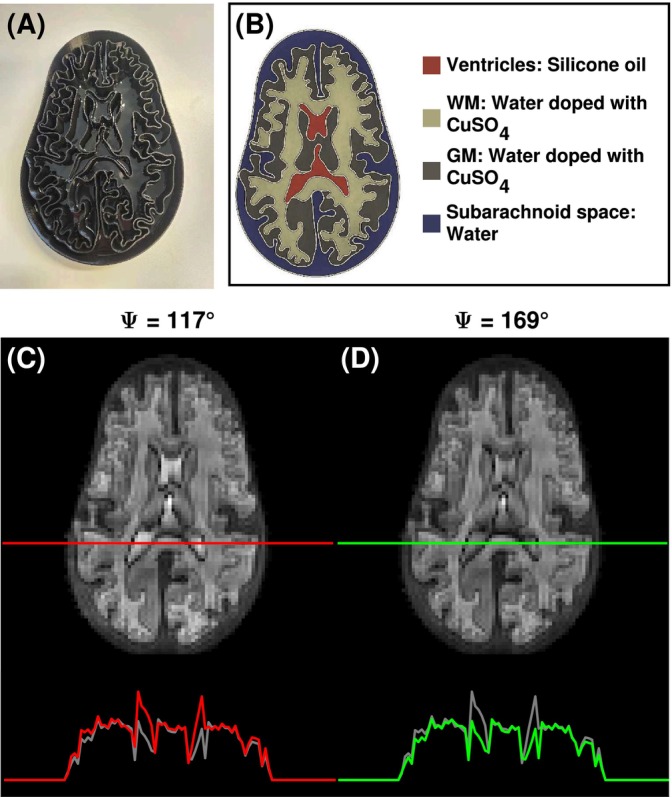
In vitro measurement of a steady‐state FLASH sequence of a 3D‐printed brain structure (A, B). The ventricles are filled with silicone oil, GM and WM with water doped with CuSO_4_, the subarachnoid space with water. The phantom parameters are for silicone oil (PD ≈ 1.0, T_1_ ≈ 630 ms, T_2_ ≈ 125 ms, D ≈ 0.03 × 10^−3^ mm^2^/s), GM (PD ≈ 0.35, T_1_ ≈ 140 ms, T_2_ ≈ 90 ms, D ≈ 2.3 × 10^−3^ mm^2^/s) and WM (PD ≈ 0.4, T_1_ ≈ 100 ms, T_2_ ≈ 50 ms, D ≈ 2.0 × 10^−3^ mm^2^/s). Two different phase difference increments Ψ = 117° (C) and 169° (D) are measured. Line plot is shown for both measurements in red (Ψ = 117°) and green (Ψ = 169°). Sequence parameters are α = 5°, TE = 1.23 ms, TR = 2.72 ms, FOV = 220 × 220 × 20 mm^3^, matrix size of 100 × 100 with gradient spoiler in read direction with a moment corresponding to 0.5 times the maximum k‐space gradient moment, partial Fourier factor of 6/10 in read direction, bandwidth of 2000 Hz/pixel.

The contrast changes can also be experimentally observed using silicone oil with a low diffusion coefficient of approx. 0.03 × 10^−3^ mm^2^/s. In the silicone oil filled compartment (corresponding to the ventricles), a signal increase is observed for ψ = 117° compared to ψ = 169° (Figure 4), whereas the remaining compartments show no significant change.

## 4 Discussion

We show that even in the case of constant gradient‐spoiling and quadratic phase cycling the refocusing and stimulation part of the excitation pulses can bring back “spoiled” transverse magnetization, refocusing both the T'_2_‐ and gradient‐dephasing. Thus, there is a relaxation‐based spoiling process not within one TR, but over several TRs that depends on relaxation processes such as T_2_ decay and gradient‐induced diffusion decay, a new spoiling category: a “multi‐TR‐relaxation‐spoiling”. These signals contribute to the FLASH sequence in the steady state, depending on the actual phase difference increment Ψ.

Surprisingly, many quadratic phase cycling in use (Ψ = 50°, 84°, 117°), that are meant to be ideally RF‐spoiled, actually require this additional “multi‐TR‐relaxation‐spoiling”, while less‐used values (Ψ = 115.4°, 169°) can achieve efficient RF‐spoiling without this process [6]. A quadratic phase cycling with phase difference increment of 169° preserves the T_1_w contrast for small diffusion values and was already suggested by Leupold et al. [6] and Yarnykh et al. [14]. We could show by phase graph analysis (Figure 2) that more higher states cancel each other more efficiently for this setting. However, also these quadratic phase cyclings show deviations from the ideal transient signal decay (Figure 1c), which can be reduced by leaving the quadratic phase cycling regime [16].

The two types of quadratic phase cycling (Ψ = 50°, 84°, 117°) and (Ψ = 115.4°, 169°) work equally well in healthy in vivo tissue, but only due to the coincidence that either T_2_‐decay (WM, GM) or diffusion (CSF) processes diminish recurring echo pathways. For imaging scenarios of oils or other tissues with lower diffusion (e.g., in pore/capillary materials with very low diffusion lengths [19, 20, 21]), such as used in studies involving phantoms or contrast media [6, 22, 23] the contrast change is relevant. The contrast change of silicone oil for different phase difference increments was already shown by Leupold et al. [5] which we could confirm using a 3D‐printed phantom filled with silicone oil. In clinical routine, silicone oil is routinely used in the treatment of retinal detachment. In rare cases, silicone oil may migrate along the optic nerve into the cerebral ventricular system [44, 45, 46]. A contrast change of silicone oil can occur for different phase difference increments. Awareness of this phenomenon is therefore important to avoid misinterpretation of other intracranial pathologies.

In the time of growing numbers of MR simulations, synthetic images are increasingly used as training data. There, the contrast change of simulated FLASH images gets crucial. Omitting diffusion from simulations can lead to qualitative contrast inversions, misrepresenting tissue properties and potentially compromising downstream applications such as segmentation, classification, or parameter mapping. As an example, we built a segmentation network, which fails for in vivo data if diffusion is not considered during training data generation.

## 5 Conclusion

FLASH sequences produce T_1_w contrast under the assumption of ideal spoiling. However, we showed that gradient‐spoiling and most RF‐spoiling schemes alone are insufficient to achieve ideal spoiling conditions.

In practice, the FLASH T_1_‐weighting contrast is a very complex interplay of gradient‐ and RF‐spoiling, and T_2_ and diffusion processes over multiple TRs (“multi‐TR‐relaxation‐spoiling”): Sufficient T_2_ relaxation and diffusion‐induced dephasing effects suppress higher‐order echo pathways, resulting in predominantly T_1_‐weighted contrast. In the absence of this spoiling mechanism, the FLASH steady‐state signal significantly depends on T_2_. As a result, CSF, with its long T_2_ values, can have a higher signal than WM, breaking the T_1_‐weighting in simulations without diffusion effects.

Neglecting diffusion in simulation could lead to unrealistic contrast and misrepresentation of tissue properties, potentially compromising downstream applications such as segmentation, classification, or parameter mapping when simulated training data is used. Only specific quadratic phase cycling settings (e.g., 169°) can be seen as ideal RF‐spoiling, whereas others lead per se to both T_2_ and diffusion weighting in steady‐state FLASH MRI. In healthy in vivo tissue, this effect is typically quenched by sufficiently high diffusion and T_2_ relaxation, but could be observed in low‐diffusivity media like silicone oil.

## Funding

We are thankful for support by the Competence Network for Scientific High Performance Computing in Bavaria (KONWIHR), the German Federal Ministry for Research, Technology and Space (BMFTR, Grant No. 16SV9585), the German Research Foundation (DFG, Grant No. 500888779/RU5534), and the d.hip Campus – Bavarian AIM.

## Conflicts of Interest

Deepak Charles Chellapandian, Fabian Wagner and Rainer Schneider are employees of Siemens Healthineers AG.

## Supporting information


**Figure S1:** Phantom maps for proton density (a), T_1_ (b), T_2_ (c), and D (d). In each quadrant one tissue parameter of the phantom was modified within a shape of the respective label (PD, T1, T2, or D).
**Figure S2:** Steady‐state contrast of FLASH sequence for different scaling factors of diffusion (1% and 100%) and R2 (20% and 100%) for phase difference increment of 84° (A) and 169° (B). Sequence parameters are α = 8°, TE = 2.0 ms, and TR = 4.0 ms. Steady‐state contrast of FLASH sequence for increasing FA (α = 0.8°/8°) and TR (TR = 0.04 ms/4 ms) for phase difference increment of 84° (C) and 169° (D). In each square one parameter of the phantom was modified within a shape of the respective label (PD, T1, T2, or D).
**Figure S3:** Steady‐state contrast for FLASH sequence with phase difference increment of 84° (A) and 169° (B) for increasing isochromat number. Sequence parameters are α = 8°, TE = 2.0 ms, and TR = 4.0 ms. Diffusion is set to 0 × 10^−3^ mm^2^/s for all simulations. T_2_‐map of the phantom is scaled by 10% in the left part of the brain to visualize the influence of T2 (see T_2_ map in Supporting Information Figure S4D).
**Figure S4:** Steady‐state contrast for FLASH sequence with phase difference increment of 84° (A) and 169° (B) for increasing simulation accuracies. Sequence parameters are α = 8°, TE = 2.0 ms, and TR = 4.0 ms. Diffusion is set to 0 × 10^−3^ mm^2^/s for all simulations. T_2_ map of the phantom is scaled by 10% in the left part of the brain to visualize the influence of T_2_ (D). Difference between MR image simulated with accuracy 1e^−8^ and 1e^−1^ for a phase difference increment of 84° (C) and 169° (E) shows T_2_ contrast. For Ψ = 169°, the difference is scaled by a factor of 10.
**Figure S5:** Simulated training data (A) for segmentation network of (Figure 3). Additional noise and/or bias fields are not shown. Contrast change for Ψ = 117° and no diffusion effects in simulation is clearly visible in regression plots (B and C).

## Data Availability

The data that support the findings of this study are available from the corresponding author upon reasonable request.
