# Supplementary material for: T1‐weighting in Steady‐State FLASH MRI–Diffusion Is Not Only Supportive but Mandatory for the Contrast
Source: Magn Reson Med. 2026 Jun 21;96(4):1789–97. doi: 10.1002/mrm.70443 (PMC13421012; doi:10.1002/mrm.70443)
Supplement: Supplementary file 1 — Figure S1: Phantom maps for proton density (a), T1 (b), T2 (c), and D (d). In each quadrant one tissue parameter of the phantom was modified within a shape of the respective label (PD, T1, T2, or D). Figure S2: Steady‐state contrast of FLASH sequence for different scaling factors of diffusion (1% and 100%) and R2 (20% and 100%) for phase difference increment of 84° (A) and 169° (B). Sequence parameters are α = 8°, TE = 2.0 ms, and TR = 4.0 ms. Steady‐state contrast of FLASH sequence for increasing FA (α = 0.8°/8°) and TR (TR = 0.04 ms/4 ms) for phase difference increment of 84° (C) and 169° (D). In each square one parameter of the phantom was modified within a shape of the respective label (PD, T1, T2, or D). Figure S3: Steady‐state contrast for FLASH sequence with phase difference increment of 84° (A) and 169° (B) for increasing isochromat number. Sequence parameters are α = 8°, TE = 2.0 ms, and TR = 4.0 ms. Diffusion is set to 0 × 10^−3^ mm2/s for all simulations. T2‐map of the phantom is scaled by 10% in the left part of the brain to visualize the influence of T2 (see T2 map in Supporting Information Figure S4D). Figure S4: Steady‐state contrast for FLASH sequence with phase difference increment of 84° (A) and 169° (B) for increasing simulation accuracies. Sequence parameters are α = 8°, TE = 2.0 ms, and TR = 4.0 ms. Diffusion is set to 0 × 10^−3^ mm2/s for all simulations. T2 map of the phantom is scaled by 10% in the left part of the brain to visualize the influence of T2 (D). Difference between MR image simulated with accuracy 1e−8 and 1e−1 for a phase difference increment of 84° (C) and 169° (E) shows T2 contrast. For Ψ = 169°, the difference is scaled by a factor of 10. Figure S5: Simulated training data (A) for segmentation network of (Figure 3). Additional noise and/or bias fields are not shown. Contrast change for Ψ = 117° and no diffusion effects in simulation is clearly visible in regression plots (B and C). [file MRM-96-1789-s001.docx]

# Supporting Information

To investigate the actual contrast-weighting we use a modified simulation where the brain tissue parameters are altered: in each brain quadrant a different tissue parameter was modified within a shape of the respective label (PD, T1, T2, or D), see Figure 3. In these letter shaped areas, T2 was increased by 2s, PD was increased by 0.5, T1 was increased by 4s, and D was increased by 6x10^-3^ mm^2^/s. For these altered values, the resulting T2-map and the D-map was additionally scaled, so we can study the contrast for different regimes of T2 and D. If a label occurs in a resulting simulated image, this indicates the corresponding weighting.*Figure S1: Phantom maps for proton density (a), T_1_ (b), T_2_ (c), and D (d). In each quadrant one tissue parameter of the phantom was modified within a shape of the respective label (PD, T1, T2, or D).*

Figure S2Aa shows, for low R_2_ relaxation effects and low diffusion effects the FLASH contrast depends in addition to T_1_ and PD also on T_2_ and diffusion. This scenario corresponds to inefficient “multi-TR-relaxation-spoiling”. If only R_2_ relaxation is increased to realistic values (Figure S2Ab), we have the same weighting, and still T_2_-weighting and thus also still the higher intense CSF. When only D is increased to realistic values, but R_2_-relaxation remains low (Figure S2Ac), we see that the T_2_-weighting vanishes and the image looks almost as expected. When both R_2_-relaxation and diffusion are at realistic high values (Figure S2Ad), the diffusion-weighting is suppressed, and the expected T_1_-weighting is visible, which notably also has PD contribution. For a phase difference increment of 169° even small diffusion values do not lead to T_2_-weighting or D-weighting (Figure S2B).

We can conclude, that in the in vivo regimes the TR-spoiling is efficient for FLASH sequences for clinical TR values. There, the contrast is T_1_-weighted, but the general FLASH contrast depends on the R_2_ and D-regimes. Per se, steady-state FLASH sequences can be diffusion and T_2_-weighted.

Not changing the phantom parameter, but the sequence parameter, the contrast composition is generally interesting when thinking of order of magnitudes different, especially shorter TR (Figure S2C). Using a phantom with realistic tissue parameters and a quadratic phase cycling with phase difference increment of 84°, a very short TR and corresponding Ernst angle leads to D-weighting (Figure 5Ca). The dominant contrast is PD-weighted and no T_1_-weighting is visible because of the very short TR = 0.04 ms. If we leaving the regime of the Ernst angle, we see a strong influence of T_2_ and D on the contrast (Figure 5Cb). For small flip angles and realistic TR of 4 ms, PD-weighting is still dominant (Figure 5Cc). Figure 5Cd shows the original, expected T_1_-weighted contrast. For smaller TR and FA, a quadratic phase cycling with phase difference increment of 169° also outperforms 84° as shown in Figure S2D.

**(B)**

**Ψ = 169°**

**Ψ = 84°**

**(A)**

**(C)**

**Ψ = 169°**

**Ψ = 84°**

**(D)**

Figure S2: Steady-state contrast of FLASH sequence for different scaling factors of diffusion (1% and 100%) and R2 (20% and 100%) for phase difference increment of 84° (A) and 169°(B). Sequence parameters are α = 8°, TE = 2.0 ms, and TR = 4.0 ms. Steady-state contrast of FLASH sequence for increasing FA (α = 0.8° / 8°) and TR (TR = 0.04 ms / 4 ms) for phase difference increment of 84° (C) and 169°(D). In each square one parameter of the phantom was modified within a shape of the respective label (PD, T1, T2, or D).

Isotropic diffusion effects are already considered in the isochromat simulation Koma [C. Castillo‐Passi, R. Coronado, G. Varela‐Mattatall, C. Alberola‐López, R. Botnar, and P. Irarrazaval, “KomaMRI.jl: An open‐source framework for general MRI simulations with GPU acceleration,” Magn. Reson. Med., vol. 90, no. 1, pp. 329–342, Jul. 2023, doi: 10.1002/mrm.29635.], JEMRIS [T. Stöcker, K. Vahedipour, D. Pflugfelder, and N. J. Shah, “High‐performance computing MRI simulations,” Magn. Reson. Med., vol. 64, no. 1, pp. 186–193, Jul. 2010, doi: 10.1002/mrm.22406.] or in phase distribution graph based simulation [J. Endres, S. Weinmüller, H. N. Dang, and M. Zaiss, “Phase distribution graphs for fast, differentiable, and spatially encoded Bloch simulations of arbitrary MRI sequences,” Magn. Reson. Med., vol. 92, no. 3, pp. 1189–1204, Sep. 2024, doi: 10.1002/mrm.30055.] as used herein. An isochromat simulation without diffusion is done in the Supporting Information Figure S3 and shows the same contrast change. A high number of isochromats is necessary for high T_2_ values to occupy the higher states. In general, isochromat Bloch simulators struggle with intra-voxel effects like diffusion, since this effect requires many spins per voxel, which increases simulation time. In phase graph simulations diffusion effects can very efficiently be expressed by specific diffusion weightings of individual magnetization pathways [M. Weigel, S. Schwenk, V. G. Kiselev, K. Scheffler, and J. Hennig, “Extended phase graphs with anisotropic diffusion,” J. Magn. Reson., vol. 205, no. 2, pp. 276–285, Aug. 2010, doi: 10.1016/j.jmr.2010.05.011.]. In a phase distribution graph simulation higher diffusion leads to a faster decay of higher-order echoes. If the signal of these states falls below the predefined accuracy threshold, they no longer need to be simulated, resulting in a faster overall simulation computation. However, higher echoes can have a small amplitude but can accumulate and significantly alter image contrast. PDG simulations in this case must be executed with high accuracy, and corresponding low latent signal thresholds are necessary as shown in the Supporting Information in Figure S4. An accuracy value of 1 means that only the state with the highest signal contribution is considered. A noticeable contrast change occurs particularly when increasing accuracy from 1e-2 to 1e-3, and again from 1e-4 to 1e-5 for Ψ = 84°. These findings indicate that, although higher-order states exhibit very small amplitudes, their cumulative contribution can lead to measurable contrast differences. To ensure sufficient simulation accuracy, at least 1e-5 accuracy is required, although this also depends on parameters such as TR, flip angle, and T_2_​. The clear T_2_ contribution can be seen in the difference between high and low accurate simulation in Figure S4C. Interestingly, already an accuracy of 1e-1 predicts similar contrast as simulating with an accuracy of 1e-8 for a phase difference increment of 169°. Knowing from the latent plot in Figure 2C in the main manuscript, also higher states exist for this phase difference increment. However, contributions from higher states cancel out and, in contrast to a phase difference increment of 84°, do not sum up to a notable contrast change.

Figure S3: Steady-state contrast for FLASH sequence with phase difference increment of 84° (A) and 169° (B) for increasing isochromat number. Sequence parameters are α = 8°, TE = 2.0 ms, and TR = 4.0 ms. Diffusion is set to 0x10^-3^ mm^2^/s for all simulations. T_2_-map of the phantom is scaled by 10% in the left part of the brain to visualize the influence of T_2_ (see T_2_ map in Supporting Information Figure S4D).

Figure S4: Steady-state contrast for FLASH sequence with phase difference increment of 84° (A) and 169° (B) for increasing simulation accuracies. Sequence parameters are α = 8°, TE = 2.0 ms, and TR = 4.0 ms. Diffusion is set to 0x10-3 mm2/s for all simulations. T2 map of the phantom is scaled by 10% in the left part of the brain to visualize the influence of T2 (D). Difference between MR image simulated with accuracy 1e-8 and 1e-1 for a phase difference increment of 84° (C) and 169° (E) shows T2 contrast. For Ψ = 169°, the difference is scaled by a factor of 10.


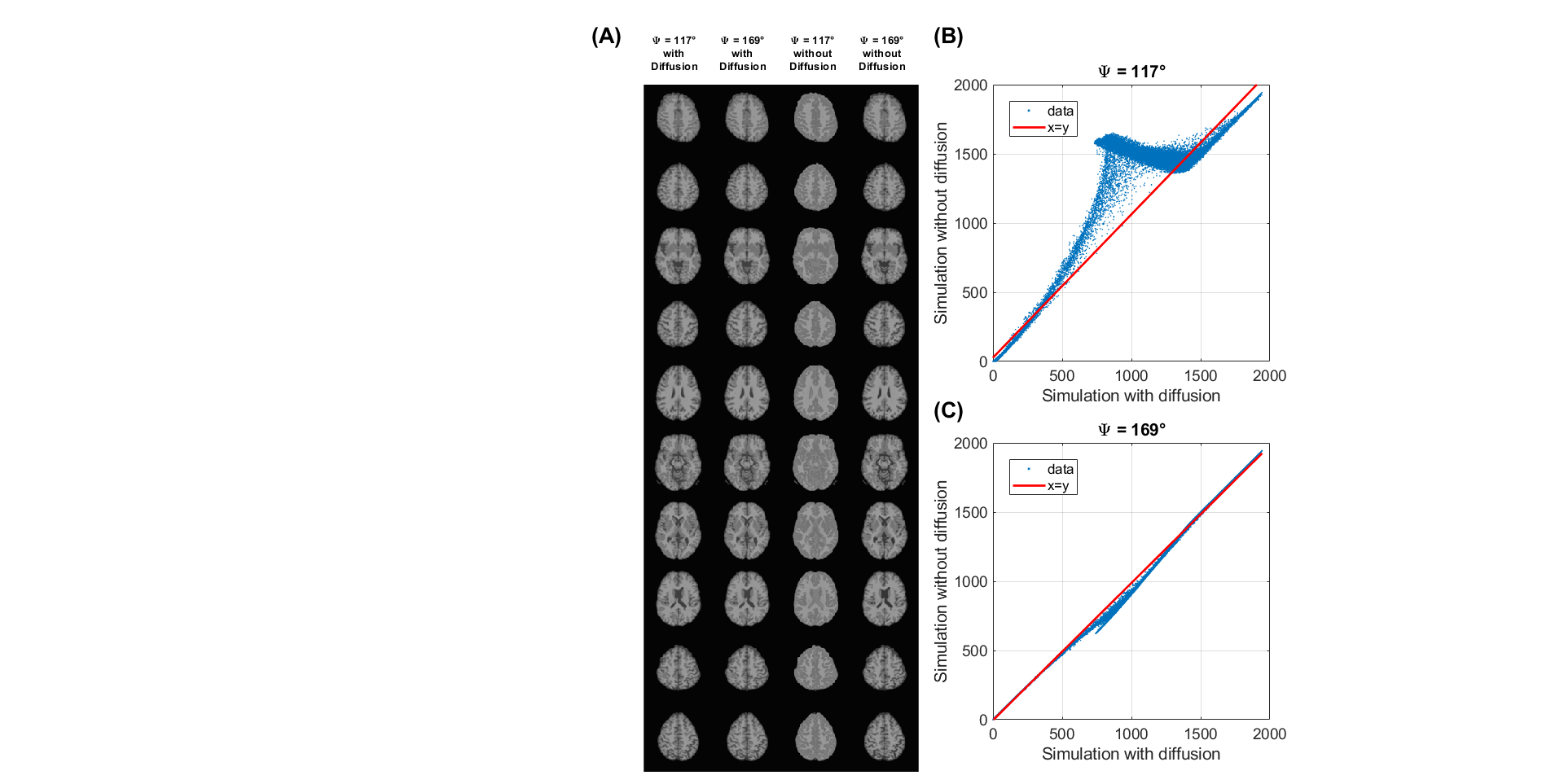


Figure S5: Simulated training data (A) for segmentation network of Figure 3. Additional noise and/or bias fields are not shown. Contrast change for Ψ = 117° and no diffusion effects in simulation is clearly visible in regression plots (B and C).
